# Progress in Medicine

**Published:** 1902-03-08

**Authors:** 


### PROGRESS IN MEDICINE.

### RHEUMATISM.

CONTRASTING the diseases Rheumatism and the fusiform "rheumatic" arthritis, Garrod[1] points out the elements of dissimilarity and holds out the possibility of the bacteriologist producing evidence to show that the diseases are essentially different, or that conditions widely differing in their clinical features may result from the same micro-organism.

Whatever may be the causation of these various joint lesions, he considers that the first element in treatment should be to remedy as far as possible any debilitating influences constantly present. Such influences may be encouraging a diseased condition arising from other causes. He instances profuse menorrhagia, dyspepsia, and unhealthy conditions of the mouth or gums. Low diet should be avoided as being more calculated to promote than retard the disease, and so does more harm than good. In those who have been used to them, stimulants seem to do good rather than harm. Tonics, such as cod-liver oil, iron, and arsenic, should be given; anodynes for pain, and fresh air and regulated exercise. Massage and passive movements often do more harm than good, but as far as spas and baths are concerned, the douche-massage as practised at Aix-les-Bains seems attended with better results than immersion baths. In the form he terms the crippling or ankylosing type he has seen no particular benefit result from any form of treatment.

While Armstrong[2] urges that in cases of arthritis there may be very different causal conditions such as in eight instances he quotes, but one factor common to all, viz., that they had been lowered in general health through nerve strain, worry, or anxiety, and so a bacterial theory is not supported, Cave[3], on the other hand, considers that such a theory is not disproved. He quotes a case where a condition of osteoarthritis of typical character was coincident with and dependent upon an extensive ulceration of the rectum. The two conditions improved simultaneously. He asks if this was not a case where a septic infection was producing an osteoarthritis; and so "rheumatoid arthritis" be the similar expression of various infective agents. He considers the vital question to be, when all known infective agencies are put aside, is there a class due to a specific infection which manifests itself only in connection with a chronic progressive arthritis? However, it may be that even if the exciting cause has been removed, the process started may still continue.

Armstrong suggests that in all cases of arthritis there is more or less interference with the nerve nutrition of the affected joint or joints: but in many of the rheumatic or gouty forms the trophic factor is present in small degree only, and are therefore the more amenable to well-directed treatment. When, however, the trophic factor predominates the cases prove more refractory. His treatment is aimed at treating the basic cause—rheumatism or gout; eliminating any cause of reflex irritation; building up the health of patients, especial attention being paid to the nervous system and the spinal cord in particular, the most valuable agent being the electric bath; a generous diet, always leaning towards an abundance of butcher's meat to the exclusion of carbohydrates; and prolonged administration of liq. auri et arsen. bromid.

The "acute rheumatoid arthritis" is considered by Luff[4] to be of bacterial origin, and in the treatment of it he believes in generous diet, radiant-heat baths, and prolonged administration of pot. iod. with one of the guiacol preparations. But Caldwell[5] considers that potassium iodide is too lowering and too liable to produce anæmia. He recommends rest as the most important part of the treatment, with passive movements and massage. Early recognition in this is as valuable as in tuberculosis.

Herringham[6] looks more to the points of similarity rather than those of difference in Still's three forms of osteoarthritis in the young, and explains the difference between the young and the old forms as being due to the variability of resistance in the young and old tissues.

In arthritis due to injury—"traumatic arthritis"—Arbuthnot Lane[7] states that the bruising of the cartilage or vascular osseous tissue beneath it may be so great as to lay open the opportunity for tubercle to enter. A shock to a young joint, if greater than it can stand, may so damage the cartilage that it may never be restored again. If the joint be kept at rest then the destructive process may be prevented, but if not then the cartilage becomes gradually absorbed. After 40 to 50, and in labouring classes earlier, the bruised cartilage, instead of being restored, becomes gradually removed, leaving an excavated surface which, if the damage be great may proceed to eburnation. Owing to this a chronic inflammation of the synovial membrane occurs, which leads to thickening, excessive secretion of synovial fluid, and the joint becomes unstable. To meet this lips or edges of bone form and ossification of the synovial fringes and ligaments ensues with deposits of bone in cavities, *e.g.*, olecranon and coronoid forsæ, limiting the range of movement but increasing security. These are the changes which are often miscalled those of "osteoarthritis."

1 Brit. Med. Journ., Oct. 12. 2 Ibid. 3 Ibid. 4 Ibid. 5 Ibid. 6 Ibid. 7 Clin. Jour., Sept. 25.

